# Validation of the Malay version of the Multidimensional Scale of Perceived Social Support (MSPSS-M) among patients with cancer in Malaysia

**DOI:** 10.1371/journal.pone.0293698

**Published:** 2023-11-21

**Authors:** Wenjun Song, Nor Shuhada Mansor, Nurul Izzah Shari, Nizuwan Azman, Ruiling Zhang, Mohammad Farris Iman Leong Bin Abdullah

**Affiliations:** 1 The Second Affiliated Hospital of Xinxiang Medical University, Xinxiang, Henan, People’s Republic of China; 2 Department of Community Health, Advanced Medical and Dental Institute, Universiti Sains Malaysia, Kepala Batas, Pulau Pinang, Malaysia; 3 School of Human Resource Development and Psychology, Faculty of Social Sciences and Humanities (FSSH), Universiti Teknologi Malaysia, Skudai, Johor, Malaysia; 4 Bioinformatics and Biostatistics Section, Advanced Medical and Dental Institute, Universiti Sains Malaysia, Kepala Batas, Pulau Pinang, Malaysia; University College Dublin, IRELAND

## Abstract

**Background:**

The well-being and adaptive functioning of patients with cancer depend on their perception of social support. To accurately assess and understand the impact of social support in a diverse population, validated measurement tools are essential. This study aimed to adapt and validate the Malay version of the Multidimensional Scale of Perceived Social Support (MSPSS-M) among patients with cancer in Malaysia.

**Methods:**

A total of 346 cancer patients with mixed disease types were recruited and completed the socio-demographic and clinical characteristics questionnaire and the MSPSS-M. The MSPSS-M was assessed for internal consistency, construct validity, face, content, convergent, discriminant validity, and confirmatory factor analyses.

**Results:**

The MSPSS-M and its three domains demonstrated good internal consistency with Cronbach’s α ranging from 0.900 to 0.932. Confirmatory factor analysis (CFA) of the MSPSS-M supported the three-factor model of the original English version of the MSPSS. The MSPSS-M also exhibited good convergent validity and discriminant validity.

**Conclusion:**

The MSPSS-M demonstrates favorable psychometric properties among patients with cancer in Malaysia. The validation of the MSPSS-M provides a culturally adapted and linguistically valid instrument to assess perceived social support among Malay-speaking patients with cancer in Malaysia.

## Introduction

Cancer is a debilitating disease affecting millions worldwide, posing significant physical, emotional, and social challenges. The prevalence of cancer is on the rise in Malaysia, whereby the total number of cancer cases per year increased from 5410 cases per year in 2010 to 13345 cases per year for breast cancer, while there was an increase from 4403 cases per year to 9679 cases per year for lung cancer. In 2018, the number of deaths attributed to cancer was 26,395 cases, comprising more than 50% of mortality from non-communicable diseases [1]. The experience of cancer diagnosis and treatment often leads to heightened distress and diminished well-being, making the presence of social support crucial for patients’ overall coping and adjustment [2].

Social support, defined as the perception of assistance, care, and understanding from others, is vital in alleviating the psychosocial burden associated with cancer. Social support can act as a buffer against the stress brought about by stressful events in life [3, 4]. Social support is an interactive and interpersonal construct that includes how well a social network can provide adequate support in the form of emotional (good support to enable one to feel love and have someone to trust), informational (support in the form of giving advice and guidance) and instrumental (availability of immediate help) context to an individual [5]. Adequate social support can keep patients with cancer mentally healthy by alleviating the negative effects of anxiety, depression, stress, and loneliness and adapting to the negative impact of the clinical complications of cancer and its treatment [6, 7]. Moreover, adequate social support may also enhance the quality of life of patients with cancer [8]. Therefore, many researchers regard social support as an essential indicator of well-being and develop various measurement tools to evaluate social support among patients with cancer.

In essence, the source of social support that supports a cancer patient is critical to determine whether the individual will perceive the social support received as positive or otherwise. Patients with cancer commonly regard family, friends, and significant others (such as their spouse or partner) as important sources of social support. They utilize this social support as their coping mechanism against the difficulty of living with cancer and its treatment [5]. Using validated measurement tools is imperative to accurately assess and understand social support’s impact on cancer patients. One widely used instrument is the Multidimensional Scale of Perceived Social Support (MSPSS), developed by Zimet, Dahlem, Zimet, and Farley (1988). The MSPSS includes three different forms of perceived social support, reflecting its multifaceted aspects, including family, friends, and significant others. It has received considerable validation and has been used with various demographics, proving its reliability as a measuring tool and usefulness in evaluating perceived social support [9]. The scale’s original version had a three-factor structure with adequate internal consistency (Cronbach’s α = 0.88) and stability (intraclass correlation coefficient = 0.85 after three months) [10–12]. MSPSS has been widely utilized both locally in Malaysia and in other countries. The Arabic female version in the United States [13], the Arabic version in Lebanon [14], the French version in France [15], the Hausa version in Nigeria [16], the Korean version in South Korea [17, 18], the American and the Spanish versions in Spain [19], and the revised Russian version of the MSPSS [20] are just several examples of the 22 translations into various languages [19]. Using the numerous translated versions of the MSPSS highlights how vital the scale is for examining social support.

However, conducting cultural adaptation and validation studies is crucial to ensuring the MSPSS’s applicability and accuracy in various cultural conditions. With its unique social background and rising cancer prevalence, Malaysia is a compelling location to test the MSPSS among patients with cancer. Hence, a culturally relevant and linguistically valid tool to measure perceived social support among Malay-speaking patients with cancer in Malaysia is warranted. A group of medical students from the Faculty of Medicine, University Malaya, participated in the previous MSPSS translation by Ng et al. (2010), which included validating the MSPSS-M [21]. This was followed by factorial validation of the MSPSS-M among a cohort of psychiatric patients in Malaysia [22]. In 2017, Lee et al. adapted and validated the MSPSS-M among teachers in Malaysia [23]. Furthermore, the cultural adaptation process will ensure that the MSPSS-M adequately captures the nuances of social support within the Malay-speaking community, thus enhancing its relevance and applicability. Based on the importance of the concept of social support and the extensive use of this scale in Malaysia, this study aimed to validate the reliability and validity of the Multidimensional Scale of Perceived Social Support for patients with cancer in Malaysia.

## Methods

### Study design

This is a cross-cultural validation study of the MSPSS-M for use among patients with cancer. The MSPSS-M is a 12-item self-report questionnaire that measures perceived social support from three sources: family, friends, and significant others.

### Ethics approval

This validation study was conducted with participant recruitment and data collection from January 2023 to April 2023. The authors have obtained permission from the author of the original English version of the MSPSS and the author of the original Malay version of the MSPSS-M by email to validate the Malay version of the MSPSS for use among patients with cancer. The study received approval from the Human Research Ethics Committee of Universiti Sains Malaysia (code: USM/JEPeM/22080569) and was conducted according to the Declaration of Helsinki 1964 and its subsequent amendments.

### Study setting

The study population was patients with cancer registered at the Oncology Unit of Advanced Medical and Dental Institute (AMDI), Universiti Sains Malaysia (USM). AMDI, USM serves as a leading tertiary referral center for oncology in the northern region of Peninsular Malaysia. AMDI, USM received oncology referrals from Pulau Pinang and neighboring states, such as Perak, Kedah, and Perlis.

### Sampling method

Consecutive sampling was employed as the sampling method for this study. The research team approached patients with cancer attending the outpatient clinic, daycare, and inpatient ward of the Oncology unit at AMDI, USM. They were provided with detailed information about the study, and potential subjects were screened for inclusion criteria. The inclusion criteria consisted of (1) individuals with a confirmed cancer diagnosis based on a histopathological report, regardless of cancer type or stage; (2) individuals aged 18 years or older; and (3) individuals who were able to read and write in Malay; and (4) those who were cognitively sound and physically fit to answer the questionnaires. Those who fulfilled all the inclusion criteria were invited to participate in the study and signed the written informed consent for study participation. All the participants will be given a copy of the participant information sheet for reference. Data was presented as group data, and all participants’ anonymity of personal information was assured.

### Sample size

The required sample size for CFA was calculated using the sample size calculator for structural equation models available at https://www.danielsoper.com/statcalc/calculator.aspx?id=89. The α error = 0.05, the power = 0.8, the number of latent variables = 3, the number of observed variables = 12, and the effect size = 0.21 [21]. Therefore, a sample size of 318 subjects (including a 20% estimated dropout rate) was determined for CFA. As the sample size required for CFA was the largest among all objectives, the final sample size for this study was set at 318 subjects.

### Assessment of content and face validity

Since the MSPSS had been translated into Malay and validated in other Malaysian populations, we used the already available MSPSS-M. We adapted it for use in this study. Initially, to assess the content validity, the MSPSS-M was examined by a panel of 6 experts: a psychiatrist, two psychologists, two community health specialists, and a public health specialist [24]. They were asked to assess the relevance of each item of the MSPSS-M as assigned to their designated domain. The item-level content validity index (I-CVI) was assigned a score of 0 if the expert rated the item as "not relevant to the designated domain" and a score of 1 if the expert rated the item as "relevant to the designated domain" or "very relevant to the designated domain." Universal agreement (UA) scored 0 if at least one of the experts rated the item as "not relevant to the designated domain” and scored one if all the experts agreed on the item’s relevance (all experts rated the item as "relevant to the designated domain" or "very relevant to the designated domain"). The scale level content validity index (S-CVI/UA) was calculated by summing the UA scores of 1 and dividing them by the total number of items in the MSPSS-M [25]. A score > 0.8 indicated good content validity [26]. The average scale CVI (S-CVI/Average) was determined by summing all the I-CVI scores and dividing by the total number of items in the MSPSS-M. A score > 0.9 indicated good content validity [26–29].

To assess face validity, 20 native Malay-speaking patients with cancer were given the MSPSS-M questionnaire. They were asked to answer all the items in the MSPSS-M and comment on the semantic quality, comprehensibility of all the items and instructions given, duration of administration, and any redundancy of words and sentences in an interview. The respondents’ responses to these aspects of the MSPSS-M were coded as “not appropriate,” “appropriate,” and “very appropriate.” The panel of experts will then evaluate the comments from the respondents to check whether there was a need to omit or alter the items’ wording or sentences.

### Data collection

The participants completed the sociodemographic and clinical characteristics questionnaire and the MSPSS-M.

#### Socio-demographic and clinical characteristics questionnaire

The questionnaire collected sociodemographic and clinical characteristics such as age, gender, ethnicity, monthly household income, marital status, time since diagnosis, and types and stages of cancer..”

#### Multidimensional Scale for Perceived Social Support (MSPSS)

The MSPSS is a self-administered tool to assess perceived social support from various sources, including family members, friends, and significant others. It comprises 12 items divided into three domains. Each item is rated on a Likert scale ranging from 1 (very strongly disagree) to 7 (very strongly agree). Domain scores range from 4 to 28, while total scores range from 7 to 84. Higher scores indicate a higher level of perceived social support. The MSPSS demonstrates favorable psychometric properties, including good internal consistency with a Cronbach’s α coefficient of 0.91 [9]. The MSPSS-M has been previously validated among teachers and medical students, demonstrating good internal consistency with Cronbach’s α coefficients ranging from 0.82 to 0.94 [21, 23].

### Statistical analysis

Data analysis was conducted using the Statistical Package for Social Sciences version 26 (SPSS 26), except for CFA, which was performed using the SPSS AMOS version 26 (AMOS 26). Descriptive statistics, including sociodemographic and clinical characteristics, the mean scores for each domain, and the total score of the MSPSS, were recorded. Frequency and percentage were used to present nominal data, while mean and standard deviation (SD) were used for continuous data reporting.

The reliability of the MSPSS-M was evaluated by calculating the internal consistency of the domains and the overall MSPSS-M score using Cronbach’s α coefficient [30]. In addition, internal consistency was also evaluated using McDonald’s Omega.

In the context of assessment of factorial validity of the MSPSS-M, Exploratory factor analysis was performed for the MSPSS-M among two types of population in Malaysia, such as medical students and psychiatric patients [21–23], in which three factors were extracted and rotation of items indicated that four items were designated to each factor. Hence, for validation of the MSPSS-M among cancer patients in Malaysia in this study, it would be more crucial to perform confirmatory factor analysis to confirm the best fitting factor model and the allocation of the items to specific factors of the MSPSS-M. Hence, we decided to perform CFA instead of EFA. As for the CFA, a few factor models of the MSPSS-M were compared for the best-fit model. First order and second order CFA were performed. The criteria used to select the model of best fit included: (1) a chi-square to the degree of freedom ratio (χ^2^/df) value of ≤ 2.0 with a p-value > 0.05, (2) a goodness of fit index (GFI) of ≥ 0.9 [31], (3) a Tucker-Lewis index (TLI) of ≥ 0.95, (4) a composite fit index (CFI) of ≥ 0.95, (5) a root mean square error of approximation (RMSEA) of < 0.06, and (6) a root mean square residue (RMR) of < 0.08. Models meeting these criteria were considered to have the best fit for the data [32–34].

In addition, CFA is also used to assess the validity of the constructs, such as the convergent and discriminant validity of the MSPSS-M. Convergent validity denotes that the items that form a construct converge or have a high proportion of the variance in common. The square of the standardized regression weights (SRW) represents the extent to which the variation in the corresponding item is explained by the latent construct to which it is related. Hence, convergent validity could be determined by calculating the average variance extract (AVE), whereby [35]:

$$AVE=\frac{\sum_{i=1}^{n}\lambda_{i}^{2}}{n}$$

Where *λ* represents the factor loadings.

If the AVE of each construct is more significant than 0.5, the latent construct explains more than 50% of its variation [35]. Contrastingly, discriminant validity measures the extent of a construct to capture a phenomenon that other constructs in the model do not explain. Hence, discriminant validity is evaluated by comparing if the AVE of any of the constructs is greater than the square of the correlation between any of the constructs in the model [35].

## Results

### Participants

The socio-demographic, clinical characteristics and total MSPSS-M scores of all the participants are presented in Table 1. More than three-quarters of the participants were females (n = 267, 76.9%), and slightly more than half were middle-aged, between 26 and 60 years old (n = 282, 81.6%). A significant proportion of the participants (77.5%, n = 269) belonged to the low-income group (B40) with a monthly income of less than RM4500. Regarding clinical characteristics, approximately half of the participants (47%, n = 163) had been diagnosed with breast cancer, while more than half (55.2%, n = 191) were in the early stages of cancer, specifically stage 1 and 2.

**Table 1:** Socio-demographic and clinical characteristics of the participants.

Variables	Number of participants(n)	Percentage (%)
**Age:**		
18–25 years old	6	1.7
26–60 years old	282	81.6
> 60 years	58	16.7
**Gender:**		
Male	79	22.8
Female	267	76.9
**Ethnicity:**		
Malays	279	80.4
Others	67	19.6
**Monthly household income:**		
B40 (< RM 4,500)	269	77.5
M40 (RM 4500-RM 11000)	72	20.7
T20 (> RM 11000)	5	1.4
**Marital status:**		
Married	283	81.6
Single/divorcee/widow/widower	63	18.2
**Time since diagnosis**		
< 6 months	126	36.4
6 month- 1 year	59	17.1
> 1 year	161	46.5
**Types of cancer:**		
Breast cancer	163	47.0
Head and neck cancer	84	24.2
Others	99	28.5
**Stage of cancer:**		
Stage 1 and 2	191	55.2
Stage 3 and 4	155	44.8

### Content validity index of MSPSS-M

Table 2 presents the content validity index (CVI) results for the MSPSS-M. The item-level content validity index (I-CVI) for all items of the MSPSS-M ranged from 0.83 to 1.00, indicating good agreement among the experts. The scale-level content validity index/universal agreement (S-CVI/UA) for the MSPSS-M was 0.83, demonstrating a high level of consensus among the experts. Furthermore, the average scale content validity index (S-CVI/Average) for the MSPSS-M was 0.97, indicating excellent content validity.

**Table 2:** The content validity index (CVI) of the MSPSS-M.

Items	Expert 1	Expert 2	Expert 3	Expert 4	Expert 5	Expert 6	Expert in agreement	I-CVI	UA
Item 1	1	1	1	1	1	1	6	1	1
Item 2	1	1	1	1	1	1	6	1	1
Item 3	1	1	1	1	1	1	6	1	1
Item 4	1	1	1	1	1	1	6	1	1
Item 5	1	1	1	1	1	1	6	1	1
Item 6	1	1	1	1	1	1	6	1	1
Item 7	1	1	1	1	1	1	6	1	1
Item 8	1	1	1	1	1	1	6	1	1
Item 9	1	1	1	1	1	1	6	1	1
Item 10	1	1	0	1	1	1	5	0.83	0
Item 11	1	1	1	1	1	1	6	1	1
Item 12	1	1	1	0	1	1	5	0.83	0
The average proportion of items judged as relevant across the six experts							S-CVI/Ave:	0.97	
							S-CVI/UA:		0.83

I-CVI = item-level content validity index, UA = universal agreement, S-CVI/Ave = average of the scale-level content validity index, S-CVI/UA = average of the scale-level content validity index across universal agreement among experts

### Face validity of the MSPSS-M

During the pilot study, interviews were conducted with 20 native Malay-speaking cancer respondents to gather their feedback on the MSPSS-M. The results showed that 75% of the participants agreed that the semantic quality, comprehensibility, and duration of administration of the MSPSS-M were "appropriate." Additionally, 25% of the participants rated these aspects as "very appropriate," indicating a positive response from the participants regarding the suitability of the MSPSS-M. Moreover, all the respondents found no redundancy in the words and sentences used in the items and instructions of the MSPSS-M. Hence, no further amendment of the MSPSS-M by the panel of experts was necessary.

### Reliability of the MSPSS-M

The domains of the MSPSS-M demonstrated good internal consistency, with Cronbach’s α ranging from 0.900 to 0.929. Furthermore, the total MSPSS-M score exhibited a high level of internal consistency, with Cronbach’s α of 0.932. The domains of the MSPSS-M also showed acceptable McDonald’s omega, ranging from 0.716 to 0.793. While the McDonald’s omega of the total MSPSS-M score was 0.759, which was also acceptable. The internal consistency of the MSPSS-M is summarized in Table 3.

**Table 3:** The internal consistency of the MSPSS-M.

Domains of MSPSS-M	Cronbach’s α	McDonald’s Omega
Family Support domain	0.900	0.793
Friend support domain	0.920	0.716
Significant other support domain	0.929	0.767
Total score	0.932	0.759

### Confirmatory factor analyses of the MSPSS-M

The results of the first order CFA for the MSPSS-M indicated that the two-factor model, which combined the perceived social support from friends and significant other domains, did not demonstrate a good fit to the data (χ^2^/df = 3.066, p < 0.001; CFI = 0.929; GFI = 0.913; TLI = 0.942; RMSEA = 0.078; RMR = 0.054). The model of best fit of the MSPSS-M was a three-factor model similar to the original English version of the MSPSS (χ^2^/df = 2.000, p < 0.001; CFI = 0.971; GFI = 0.931; TLI = 0.957; RMSEA = 0.056; RMR = 0.066).

When second order CFA was performed, the main construct of social support exhibited acceptable factor loading on its three sub-construct (factor loading: perceived support from family = 0.94, perceived support from friends = 0.79, and perceived support from significant others = 0.62). Hence, this confirmed that the MSPSS-M had three sub-construct was well supported. The fit indices of the second order CFA for the three-factor model of the MSPSS-M similar to the original English version of the MSPSS were identical compared to that of the first order CFA (χ^2^/df = 2.000, p < 0.001; CFI = 0.971; GFI = 0.931; TLI = 0.957; RMSEA = 0.056; RMR = 0.066).

### The convergent and discriminant validity of the MSPSS-M

The findings on the convergent and discriminant validity of the MSPSS-M are summarized in Table 4. Regarding the convergent validity of the MSPSS-M, the AVE for the domains of the MSPSS-M were 0.793, 0.716, and 0.767, respectively. Since all the AVE of the domains were greater than 0.5, convergent validity of the MSPSS-M was achieved. For the discriminant validity, the square of the inter-construct correlations (ranging from 0.24 to 0.55) was lower than the AVE of all the domains or constructs of the MSPSS-M, indicating that the discriminant validity of the MSPSS-M was achieved.

**Table 4:** The convergent and discriminant validity of the MSPSS-M derived from the best fit 3-factor model of the MSPSS-M assessed with confirmatory factor analysis.

Indicator variables	Latent variables	Standardized loading	Square of standardized loading	The sum of squared of standardized loading	Number of indicators	AVE	Square of inter-construct correlation
**Item 1**	FaSS	0.902	0.814	3.172	4	0.793	FaSS-SSS = 0.33, FaSS-FdSS = 0.55, FdSS-SSS = 0.24
**Item 2**	FaSS	0.920	0.846				
**Item 5**	FaSS	0.900	0.810				
**Item 10**	FaSS	0.838	0.702				
**Item 3**	FdSS	0.887	0.787	2.864	4	0.716	FaSS-SSS = 0.33, FaSS-FdSS = 0.55, FdSS-SSS = 0.24
**Item 4**	FdSS	0.947	0.897				
**Item 8**	FdSS	0.759	0.576				
**Item 11**	FdSS	0.777	0.604				
**Item 6**	SSS	0.888	0.789	3.069	4	0.767	FaSS-SSS = 0.33, FaSS-FdSS = 0.55, FdSS-SSS = 0.24
**Item 7**	SSS	0.806	0.650				
**Item 9**	SSS	0.939	0.882				
**Item 12**	SSS	0.865	0.748				

## Discussion

### Reliability of the MSPSS-M

The current study has effectively adapted and validated the MSPSS-M to assess perceived social support among patients with cancer in Malaysia. Our findings revealed favorable psychometric properties of the MSPSS-M among patients with cancer in Malaysia. The reliability analysis demonstrated good internal consistency, as indicated by high Cronbach’s alpha coefficients for the overall scale and its subscales (ranging from 0.900 to 0.932). In addition, the internal consistency of the domains and total score of the MSPSS-M were also reported by McDonald’s omega, which ranged from 0.716 to 0.793, indicative of acceptable internal consistency. Cronbach’s alpha reflects an essential tau-equivalence model, meaning that item factor loadings on a single intended factor are all equal in a CFA model; the alpha-implied variance/covariance matrix restricts all covariances to be equal. For McDonald’s omega, the added advantage is that omega assumes a congeneric model, which means that factor loadings are allowed to vary in a CFA model. If items are all standardized, then the omega-implied covariance matrix allows all covariances to vary [36]. This suggests that the MSPSS-M items are interrelated and consistently measure the perceived social support construct among Malay-speaking patients with cancer. In comparison to the other Malay versions of the MSPSS, which were validated to assess perceived social support among teachers, medical students, and psychiatric patients (Cronbach’s α ranged from 0.82 to 0.94), the internal consistency of the MSPSS-M for assessing perceived social support among patients with cancer in this study was comparable [21, 23].

### Factorial validity of the MSPSS-M

In the context of the factorial validity of the MSPSS-M, CFA findings in this study supported the underlying factor structure of the MSPSS-M. The three-factor structure corresponding to family support, friend support, and significant other support was consistent with the original English version of the MSPSS and other validated and translated versions across different cultural contexts (such as the Korean, Chinese, and Swedish versions of the MSPSS) [17, 36, 37]. The fitting indices of both the first order and second order CFA of the three-factor model of the MSPSS-M were identical and had acceptable fit indices. This suggests that the MSPSS-M maintains its factorial validity when applied to patients with cancer in Malaysia, highlighting the cross-cultural applicability of the scale.

### Convergent and discriminant validity of the MSPSS-M

In the convergent validity analysis, the AVE for all the domains of the MSPSS-M based on the best fitted 3-factor model of the questionnaire in CFA was higher than 0.5, indicative of good convergent validity of the MSPSS-M. The AVE of all the domains of the MSPSS-M was also higher than the square of all the interdomain correlation coefficients, as demonstrated in the best fitted 3-factor model of the MSPSS-M, which denote discriminant validity [36]. Hence, the MSPSS-M exhibited good construct validity; all its items and domains measure what they purported to measure: family, friends, and significant other social support.

### Strengths and limitations of the MSPSS-M

Notably, regarding the strength of the validation of the MSPSS-M, it fills a critical gap in the existing literature, as it provides a culturally adapted and linguistically valid instrument to assess perceived social support among Malay-speaking patients with cancer in Malaysia. The availability of such a measure is vital for researchers and healthcare professionals to accurately evaluate the social support needs of patients with cancer and design targeted interventions to enhance their psychosocial well-being.

Despite the strengths and contributions of this study, several limitations should be acknowledged. First, the consecutive sampling method may introduce selection bias, limiting the generalizability of the findings [38]. Future research could employ a more representative and diverse sample to enhance the external validity of the MSPSS-M. Second, the cross-sectional nature of our study design precludes the assessment of test-retest reliability and responsiveness to changes over time. Future longitudinal studies are warranted to evaluate the stability and sensitivity of the MSPSS-M in capturing dynamic changes in perceived social support among patients with cancer [39]. Third, the reliance on self-report measures may introduce common method bias and subjective interpretation of social support. Future research could consider incorporating multiple informants and objective social support measures to enhance the assessment’s comprehensiveness [40]. Fourth, we did not include external scales tailored to assess convergent validity due to the lack of a Malay version instrument for measuring social support among cancer patients. Hence, we employed the assessment of convergent and discriminant validity based on AVE calculated from the best fitting factor model in CFA [35]. On contrary, recent validation study of the translation of the MSPSS assessed convergent validity by evaluating the correlation between the translated MSPSS and another instrument which measure the same construct as the MSPSS which was recommended by Campbell & Fiske (1959) [14, 41, 42]. Despite the above discrepancy, our study offers valuable insights into MSPSS-M’s psychometric properties for assessing social support in Malaysian cancer patients.

## Conclusion

In conclusion, validating the MSPSS-M among patients with cancer in Malaysia provides a reliable instrument for assessing perceived social support in this population. The favorable psychometric properties, including reliability, factorial, discriminant, and convergent validity, attest to the robustness and applicability of the MSPSS-M. The availability of the MSPSS-M holds promises for facilitating research on social support dynamics among Malay-speaking patients with cancer, facilitating clinical practice, and guiding the development of targeted psychosocial interventions to enhance the well-being and quality of life of patients with cancer.

## Supporting information

S1 Appendix (PDF)
