# Supplementary material for: Validation of the Malay version of the Multidimensional Scale of Perceived Social Support (MSPSS-M) among patients with cancer in Malaysia
Source: PLoS One. 2023 Nov 21;18(11):e0293698. doi: 10.1371/journal.pone.0293698 (PMC10662767; doi:10.1371/journal.pone.0293698)
Supplement: S1 Appendix — (PDF) [file pone.0293698.s001.pdf]

# SKALA MULTIDIMENTIONAL UNTUK SOKONGAN SOCIAL

**(The Malay version of the Multidimensional Scale of Perceived Social Support, MSPSS-M)**

*Sila baca kenyataan-kenyataan berikut. Bulatkan nombor mengikut skala di bawah*

| 1 | 2 | 3 | 4 | 5 | 6 | 7 |
|---|---|---|---|---|---|---|
| Tersangat tidak setuju | Sangat tidak setuju | Tidak setuju | Berkecuali | Setuju | Sangat setuju | Tersangat setuju |

| | | | | | | | |
|---|---|---|---|---|---|---|---|
| 1. Ada seseorang yang istimewa bersama saya bila saya dalam keadaan yang memerlukan. | 1 | 2 | 3 | 4 | 5 | 6 | 7 |
| 2. Ada seseorang yang istimewa untuk saya berkongsi kegembiraan dan kesedihan. | 1 | 2 | 3 | 4 | 5 | 6 | 7 |
| 3. Keluarga saya cuba sedaya-upaya untuk menolong saya. | 1 | 2 | 3 | 4 | 5 | 6 | 7 |
| 4. Saya mendapat pertolongan dan sokongan emosi yang saya perlukan daripada keluarga. | 1 | 2 | 3 | 4 | 5 | 6 | 7 |
| 5. Saya mempunyai seseorang yang istimewa yang benar-benar membuat saya selesa. | 1 | 2 | 3 | 4 | 5 | 6 | 7 |
| 6. Kawan-kawan saya cuba sedaya-upaya untuk menolong saya. | 1 | 2 | 3 | 4 | 5 | 6 | 7 |
| 7. Saya boleh berharap kepada kawan-kawan saya apabila sesuatu hal yang tidak baik berlaku. | 1 | 2 | 3 | 4 | 5 | 6 | 7 |
| 8. Saya boleh bercerita tentang masalah saya dengan keluarga. | 1 | 2 | 3 | 4 | 5 | 6 | 7 |
| 9. Saya mempunyai kawan-kawan yang saya boleh berkongsi kegembiraan dan kesedihan. | 1 | 2 | 3 | 4 | 5 | 6 | 7 |
| 10. Ada seseorang yang istimewa dalam hidup saya yang mengambil berat tentang perasaan saya. | 1 | 2 | 3 | 4 | 5 | 6 | 7 |
| 11. Keluarga saya bersedia untuk menolong saya membuat keputusan. | 1 | 2 | 3 | 4 | 5 | 6 | 7 |
| 12. Saya boleh bercerita tentang masalah saya dengan kawan-kawan saya. | 1 | 2 | 3 | 4 | 5 | 6 | 7 |

[1]*Ng, C.G., Amer Siddiq, A.N., Aida, S.A.,Zainal, N.Z., Koh, O.H., 2010. Validation of the Malay version of the Multidimensional Scale of Perceived Social Support (MSPSS-M) among a group of medical students in Faculty of Medicine, University Malaya. Asian Journal of Psychiatry 3, 3-6.*

[2]*Zimet, G.D., Dahlem, N.W., Zimet, S.G., Farley, G.K., 1988. The Multidimensional Scale of Perceived Social Support. Journal of Personality Assessment 52, 30-41.*
